# Peripheral Immune and Metabolic Dysregulation in Migraine, Ménière′s Disease, and Vestibular Migraine: A Single‐Cell Atlas Study

**DOI:** 10.1155/humu/2426414

**Published:** 2026-04-07

**Authors:** Mika Pan, Liyan Lu, Wenyi Song, Youfeng Xie, Guining Liang, Qingyan Wei, Qi Huang, Jingyi Zeng, Yating Lan, Chun Zou, Donghua Zou, Li Su, Qiling Wan

**Affiliations:** ^1^ Department of Neurology, The Second Affiliated Hospital of Guangxi Medical University, Nanning, Guangxi, China, gxmu.edu.cn; ^2^ Department of Neurology, Affiliated Hospital of Youjiang Medical University for Nationalities, Baise, Guangxi, China, gxyyfy.cn; ^3^ Guangxi Key Laboratory of Artificial Intelligence for Genetic Diseases of Long-Dwelling Nationalities, Baise, Guangxi, China; ^4^ Administrative Affairs Office, Wuming Hospital of Guangxi Medical University, Wuming, Guangxi, China

**Keywords:** MAPK signaling pathway, Ménière′s disease, migraine, single-cell RNA sequencing, vestibular migraine

## Abstract

**Background:**

Migraine (MI), Ménière′s disease (MD), and vestibular migraine (VM) share significant clinical and pathological similarities, particularly their link to neurological dysfunction and immune cell activity, though mechanisms remain poorly understood.

**Methods:**

We utilized a single‐cell RNA sequencing dataset of peripheral blood mononuclear cells from eight patients with MD, five patients with MI, five patients with VM, and six healthy controls. A cross‐disease cell atlas was constructed via cellular heterogeneity and dynamic cell abundance analysis. Further studies analyzed metabolic heterogeneity and performed differential expression analysis across all groups. The core regulatory pathways were identified using gene set enrichment and pathway analyses. Additionally, herbal and compound screenings related to targets in MI were performed. Pseudotime analysis was then employed to infer the evolutionary trajectories, identifying key genes associated with differentiation.

**Results:**

We identified 42,198 cells grouped into 16 clusters and classified into five cell types: T cells, B cells, dendritic cells, natural killer cells, and monocytes. Among these, T cell abundance significantly increased, whereas monocytes were essential for metabolic reprogramming. Notably, the upregulated MAPK signaling pathway was identified as the core regulatory pathway. A total of 1571 interaction pairs were screened between matched targets for herbs and compounds. T cell trajectory analysis revealed two differentiation pathways originating from CD8^+^ T cells that diverged into CD4^+^ T cells and naïve T cells. *PRKCA* was significantly upregulated during differentiation, being highly expressed in the disease groups.

**Conclusions:**

This study identified distinct immune cell patterns in MI, MD, and VM, with notable T cell imbalances. *PRKCA* was highlighted as a core regulator of disease mechanisms via the MAPK pathway, laying the groundwork for future research into therapies and disease understanding.

## 1. Introduction

Migraine (MI) is a chronic neurological condition affecting roughly 20% of the worldwide population [1]. MI is typically characterized by episodic headaches, nausea, vomiting, and sensitivity to light and sound. If left untreated, the headache may worsen, progressing to chronic MI [2]. MI is intricately associated with the occurrence of spreading depolarization, a pathophysiological phenomenon believed to play a pivotal role in the onset and progression of MI attacks [3]. Approximately 25% of patients with MI exhibit episodic vestibular symptoms, known as vestibular migraine (VM), including dizziness, vertigo, and nausea triggered by head movements [4]. Ménière′s disease (MD), presenting as episodic vertigo and MI [5], is a complex chronic inflammatory inner ear disorder marked by sensorineural hearing loss, tinnitus, or vertigo episodes associated with hearing loss [6]. Notably, significant pathophysiological similarities exist between MI, VM, and MD, emphasizing the importance of careful differential diagnosis. Therefore, optimizing diagnostic processes and developing personalized treatment plans are paramount.

Studies have suggested that immune dysregulation and inflammatory cytokine imbalance are risk factors in MI pathogenesis [7, 8, 9]. Elevated levels of proinflammatory molecules (IL‐1*β*, TNF‐*α*, and IL‐6) [10, 11] and the chemokine IL‐8 [12] in patients with MI suggest that a systemic inflammatory state may contribute to MI development. Furthermore, compared with patients with VM, those with MD may exhibit higher levels of TNF‐*α* and IFN‐*γ*, whereas levels of epithelial neutrophil‐activating peptide‐78 may be lower [13]. The differential levels of IL‐1*β*, CCL3, CCL22, and CXCL1 can effectively distinguish between VM and MD, reinforcing clinical diagnosis and treatment [14]. These results emphasize the significance of understanding immune dysregulation and inflammatory cytokines to facilitate a more accurate diagnosis and the development of targeted treatment strategies.

Single‐cell RNA sequencing (scRNA‐seq), a methodology that studies differences in gene expression at the single‐cell level, reveals the unique gene expression patterns of each cell, providing a powerful tool for studying cellular heterogeneity [15]. Although scRNA‐seq can explore immune cell heterogeneity in certain disease states by analyzing peripheral blood mononuclear cells (PBMCs) from patients with MI, there are still limitations at the broader cell‐type level and regarding deeper molecular mechanisms [16]. However, research on MI, VM, and MD is insufficient and requires further investigation. Therefore, this study is aimed at providing the first systematic analysis of the cellular dynamics and molecular mechanisms of the immune microenvironment in patients with MI, VM, and MD using scRNA‐seq technology. This fine‐scale characterization was designed to uncover disease‐specific immune heterogeneity and identify core dysregulated pathways. Building upon these mechanistic insights, we performed a network pharmacology analysis to screen for herbal compounds targeting the key genes and pathways identified from our scRNA‐seq data. Through this integrated approach, we aimed to enhance the understanding of disease pathogenesis and facilitate the development of novel therapeutic strategies.

## 2. Methods

### 2.1. Data Sources and Processing

The PBMC samples from 24 Spanish participants with no diagnosed allergies or autoimmune diseases, along with scRNA‐seq data (10× Genomics), were downloaded from the Gene Expression Omnibus database (http://www.ncbi.nlm.nih.gov/geo/) [17] based on the GPL33758 platform of GSE269117, a dataset generated in the multiomics study by López‐Escánez et al. (Immunology, 2024) [16]. This dataset included eight patients with MD, five with VM, five with MI, and six healthy controls (HCs), whereas the foundational study by López‐Escánez et al. provided the initial characterization of the GSE269117 dataset and revealed crucial insights, such as the similarity between MI and VM, the identification of two MD clusters, and the role of NK cells. Moreover, our present study uncovers the core regulatory pathways, dynamic cellular processes, metabolic alterations, and potential therapeutic targets that collectively provide a more refined and mechanistic understanding of the shared and distinct immunopathology of MI, MD, and VM.

To obtain single‐cell expression profiles, we used the Seurat R package [18] to exclude poor‐quality cells, including doublets and cells with high mitochondrial gene content. The quality control thresholds were set as follows: the lower bound for the number of detected genes per cell (nFeature_RNA > 300) ensured the exclusion of empty droplets or low‐complexity transcripts, whereas the upper bound (nFeature_RNA < 7000) served to filter out potential multiplets (doublets). A threshold for mitochondrial gene percentage (pMT < 20) was applied to remove cells undergoing apoptosis or suffering from technical stress, as a high pMT is a hallmark of compromised cellular integrity. Similarly, a threshold for hemoglobin gene percentage (pHB < 5) was used to exclude any contaminating red blood cells, which are anucleate and not of interest in immune profiling. Following quality control, the R package, DoubletFinder, was used to filter doublets, resulting in 42,198 cells of 23 samples for subsequent analyses. All samples were sourced from the same dataset (GSE269117) and underwent uniform processing; this factor was likely to have minimal impact, thus eliminating the need for batch effect analysis.

### 2.2. Analysis of Unsupervised Dimensionality Reduction and Clustering

The t‐distributed stochastic neighbor embedding (t‐SNE) [19] was established using the Seurat package to reduce dimensionality. Cell clustering was conducted in a reduced two‐dimensional space. The single‐cell atlas was visualized using the plot1cell function. Next, the FindAllMarkers function, from the Seurat package [18], was applied to determine marker genes for each cell cluster. Cell types were determined based on known markers. Additionally, the CellCycleScoring function was employed to calculate the G1, S, and G2/M phase scores for each cell, analyzing distribution characteristics of different cell types across the cell cycle. Subsequently, variations in the abundance of different cell populations across groups were investigated, and those with significantly increased abundance were identified as key pathogenic cell types.

### 2.3. Differential Expression Analysis

To identify the molecular differences between MI cells, we used the FindAllMarkers function to calculate differential expressions between MI cells. To further analyze the expression patterns of these differentially expressed genes (DEGs), we utilized the ClusterGVis package to visualize gene expression differences between MI cells. Statistical significance was set at false discovery rate (FDR)‐adjusted *p* < 0.05 using the Benjamini–Hochberg procedure.

We compared the differences in key pathogenic cell types across different disease states, and we used the FindMarkers function to analyze the DEGs between the MI, MD, VM, and HC groups. DEGs with FDR‐adjusted *p* < 0.05 using the Benjamini–Hochberg procedure were considered significant. Further, a Venn diagram analysis was performed to examine the intersecting genes across the MI, MD, and VM groups, revealing common molecular features across different disease states.

### 2.4. Functional Enrichment Analysis

To analyze the biological significance of DEGs in MI cells, we conducted gene ontology functional annotation and Kyoto Encyclopedia of Genes and Genomes (KEGG) pathway analysis applying the clusterProfiler package [20]. Additionally, we used the clusterProfiler package [20] to investigate the biological processes (BPs) and pathways of intersecting genes across the MI, MD, and VM groups.

### 2.5. Gene Set Enrichment Analysis (GSEA)

GSEA assesses gene rank distribution within a gene set to identify significantly upregulated or downregulated gene sets under specific conditions, thereby revealing potential biological mechanisms [21]. We used the clusterProfiler package [20] to establish GSEA on the DEGs from key pathogenic cell types in the MI and HC groups to explore their enrichment patterns in immune regulation and neuroinflammation‐related pathways.

Intersecting pathways were obtained, including pathways of DEGs in intercellular MI, pathways of intersecting DEGs, and pathways of GSEA in T cells between MI and HCs. Core pathways were determined as the intersecting pathways containing the largest number of functional genes.

### 2.6. Intercellular Communication Analysis

Communication networks in MI cells were analyzed using the CellChat package [22]. This analysis was based on ligand–receptor interactions to identify the communication between different cell types. Additionally, metabolic analysis of MI cells was performed using the scMetabolism package, and the results were visualized using a heat map. The contribution score algorithm was used to quantify the relative contribution of different cell types to MI′s pathology, identifying key pathogenic cell types.

### 2.7. Key Pathogenic Subtype Analysis

Nonnegative matrix factorization (NMF) effectively reduces data dimensionality, providing easy‐to‐interpret findings [23]. Using the GeneNMF package, we extracted major cell subtypes from key pathogenic cell types and further analyzed the biological significance of these subtypes.

In this study, the AddModuleScore function of the Seurat package was applied to perform gene set activity scoring that assesses the activity differences of core pathways across different disease groups, reflecting the expression intensity of these core pathways in key pathogenic cell types and across different disease subtypes.

### 2.8. Cell Trajectory Analysis

Pseudotime algorithms could conclude the differentiation trajectory of immune cells in the blood or the evolutionary process of cell types during development [24]. In this study, cell differentiation trajectories were constructed using the Slingshot package, allowing for systematic analysis of the dynamic changes in key cell types during MI′s pathology.

### 2.9. Herb and Compound Screening Related to Targets in MI

To translate our mechanistic findings into therapeutic insights, we conducted a network pharmacology analysis to identify potential herbal medicines targeting the MI‐associated genes. The TCMNP R package was used to input target genes of MI and identify therapeutic herbs and their prescriptions by the tcm_prescription function. A network diagram was constructed by connecting herbs, components, and targets, with node connectivity rankings displayed via degree_plot. Finally, a Sankey diagram randomly displayed the interrelationships among herbs, molecules, and targets using the ggsankey package (https://github.com/davidsjoberg/ggsankey).

### 2.10. Statistical Analysis

All data analyses were applied using the Bioinforcloud platform (http://www.bioinforcloud.org.cn). This platform provides a complete workflow, from data preprocessing and statistical analysis to visualization. Statistical significance was set at adjusted *p* < 0.05.

## 3. Results

### 3.1. Single‐Cell Transcriptomics Reveal the Peripheral Immune Landscape of MI, MD, and VM

**Figure 1:**
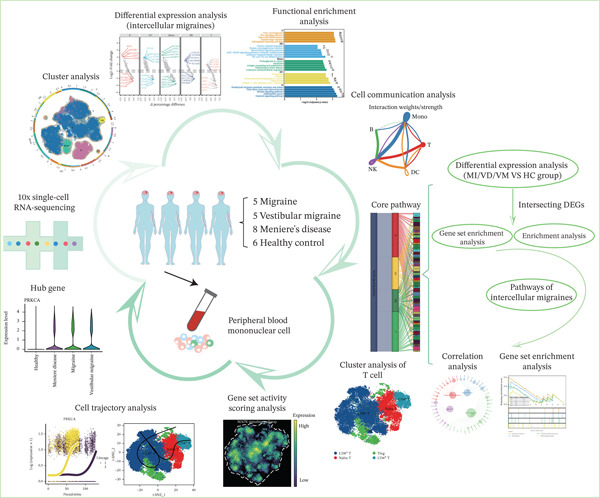
Flowchart of this study. DEGs, differentially expressed genes; MI, migraine; MD, Ménière′s disease; VM, vestibular migraine.

The workflow of this study is illustrated in the figure (Figure 1). ScRNA‐seq data were performed on PBMCs from patients with MI, MD, and VM. Following quality filtering, t‐SNE clustering analysis revealed 16 cell clusters from 42,198 cells (Figure 2a). From known marker genes, these clusters were assigned to known cell types, including monocytes (Mono), T cells, natural killer (NK) cells, dendritic cells (DCs), and B cells (Figure 2b,c). The highest proportion of these cell types was in the G1 phase, suggesting active cell cycle progression and preparation for subsequent phases of immune function (Figure 2d).

Figure 2Single‐cell immune landscape of migraine, Ménière′s disease, and vestibular migraine. (a) Clustering analysis of 42,198 cells from blood samples of healthy individuals and patients with migraine, Ménière′s disease, and vestibular migraine identified 16 cell clusters. (b) Single‐cell atlas showing the global landscape of major cell types. (c) Bubble plot of marker gene expression for different cell types. (d) Distribution of cell types across various time points. (e) Abundance changes of major cell types across different groups. DC, dendritic cell; Mono, monocyte; NK, natural killer; t‐SNE, t‐distributed stochastic neighbor embedding.

**Figure:**
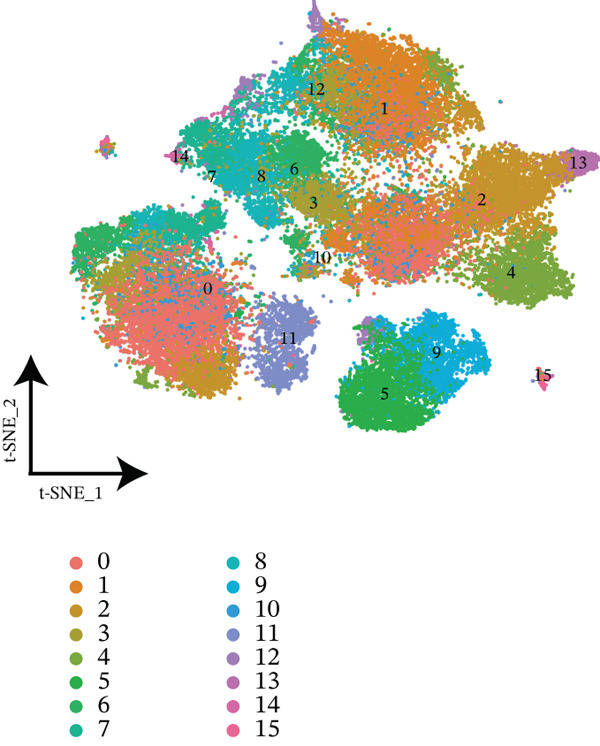
(a)

**Figure:**
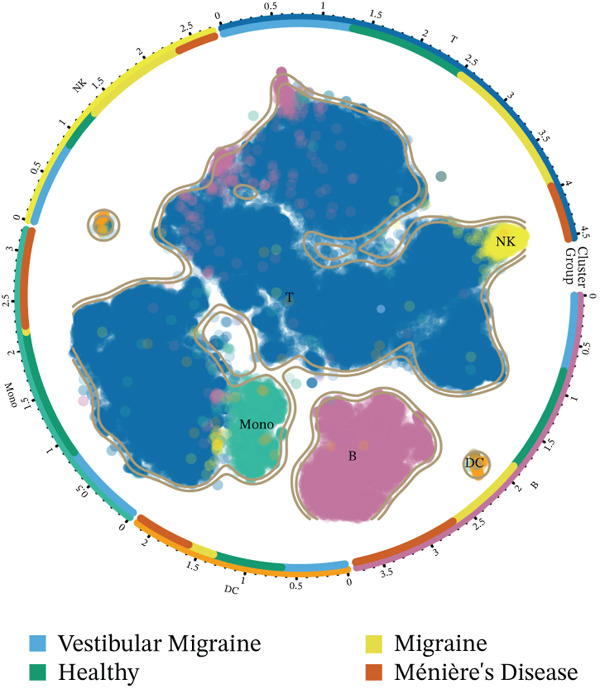
(b)

**Figure:**
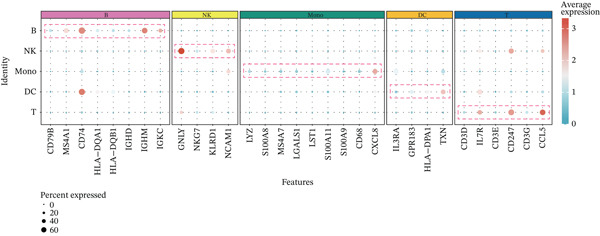
(c)

**Figure:**
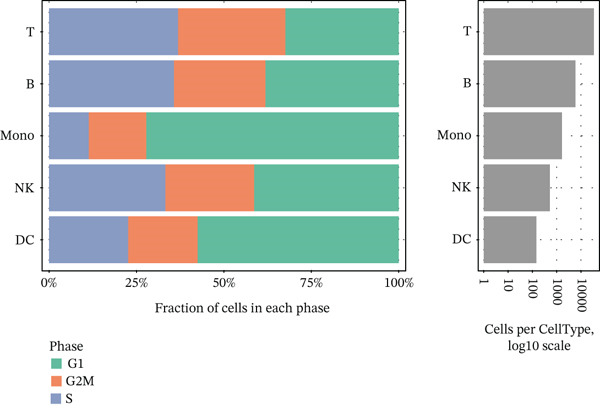
(d)

**Figure:**
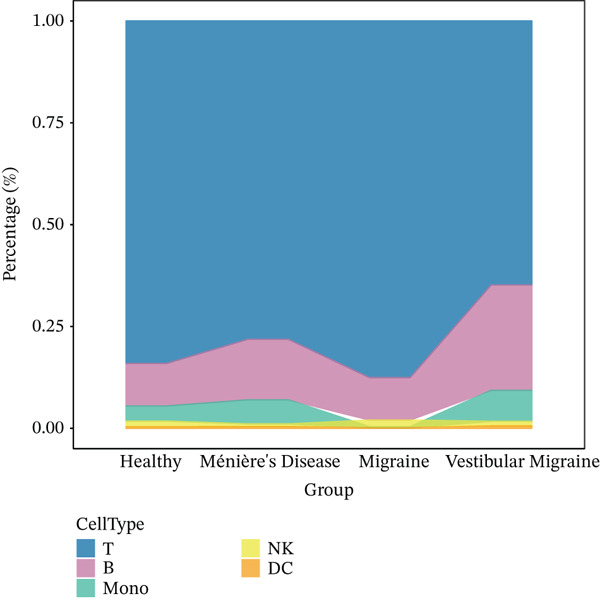
(e)

Furthermore, compared with the HC group, the MI group exhibited a significantly increased abundance of T cells, whereas a marked reduction was observed in the Mono (Figure 2e). This finding reveals that T cells undergo significant changes during headache, closely related to neuroinflammation, immune dysregulation, and immune cell interactions.

### 3.2. Single‐Cell Analysis of the Immune Microenvironment in MI: Heterogeneity, Regulation, and Metabolic Reprogramming

Previous studies have suggested that T cells, including CD4^+^ T cells and CD8^+^ T cells, are critical for immune dysregulation during MI [25]. Compared with the HC group, we found that T cells were significantly involved in the MI group, whereas their abundance was significantly reduced in the VM group (Figure 3a), suggesting that T cells may be key pathogenic cell types. In total, 4448 DEGs were identified between the MI and HC groups (Figure 3b). Among them, 384 upregulated and 654 downregulated DEGs were identified in T cells, 1049 upregulated and 177 downregulated DEGs in Mono, 710 upregulated and 393 downregulated DEGs in B cells, 192 upregulated and 228 downregulated DEGs in NK cells, and 607 upregulated and 54 downregulated DEGs in DC.

Figure 3Molecular features and intercellular communication in migraine. (a) t‐Distributed stochastic neighbor embedding clustering‐based cell clustering showing spatial distribution differences across groups. (b) The 4448 differentially expressed genes (DEGs) were upregulated and downregulated in migraine cells. Log2 fold change > 0 indicates higher expression, log2 fold change < 0 indicates lower expression. (c) Heat map showing the pathways involved in the Top 10 DEGs across different cell types. (d) Biological process analysis of DEGs in migraine. (e) Kyoto Encyclopedia of Genes and Genomes (KEGG) pathway enrichment analysis of the DEGs. (f) CellChat intercellular communication analysis identifying significantly activated ligand–receptor pairs in migraine. (g) Intercellular communication network diagram showing interaction strengths and major communication patterns. (h) Contribution analysis quantifying the relative contributions of different cell subtypes to migraine pathology. (i) Metabolic reprogramming analysis of major cell types. BP, biological processes; DC, dendritic cells; GO, gene ontology; Mono, monocyte; NK, natural killer; t‐SNE, t‐distributed stochastic neighbor embedding.

**Figure:**
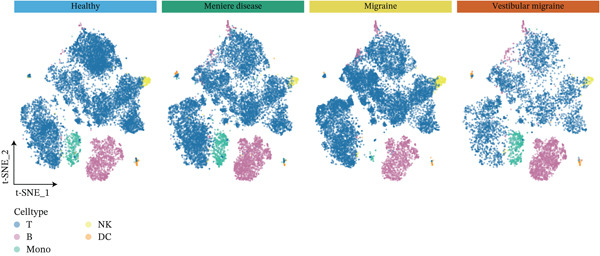
(a)

**Figure:**
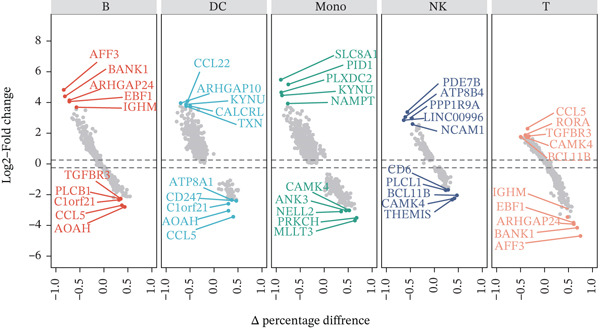
(b)

**Figure:**
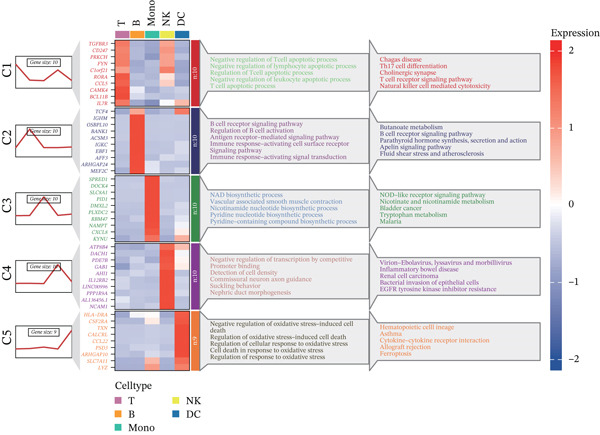
(c)

**Figure:**
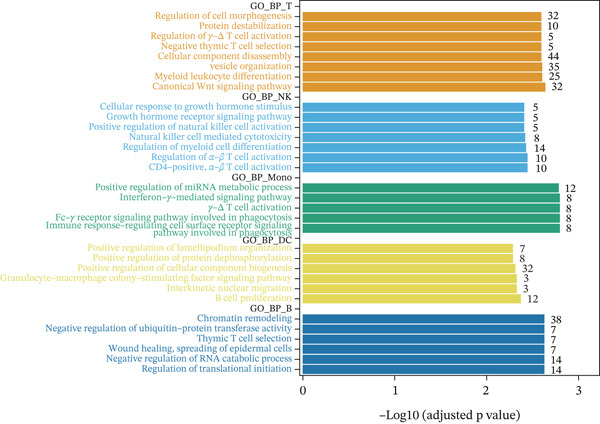
(d)

**Figure:**
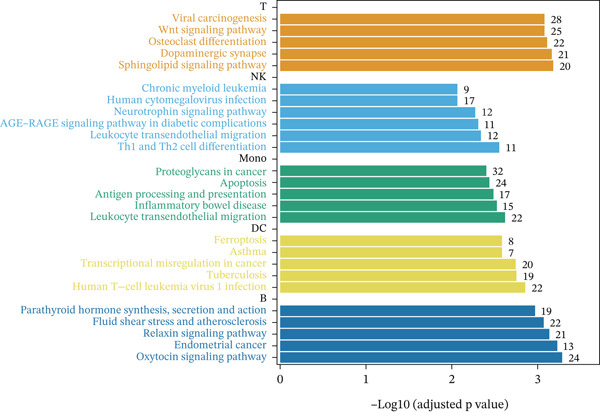
(e)

**Figure:**
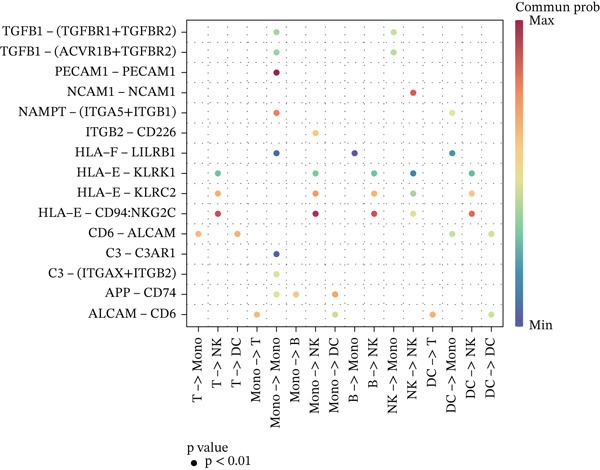
(f)

**Figure:**
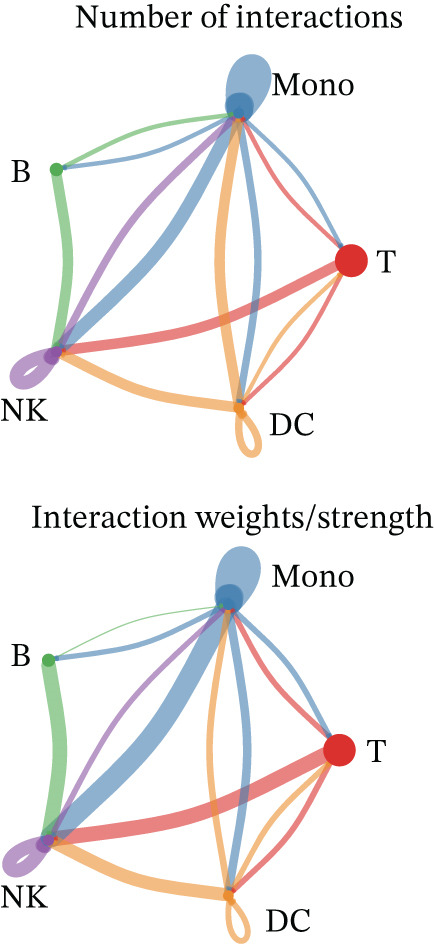
(g)

**Figure:**
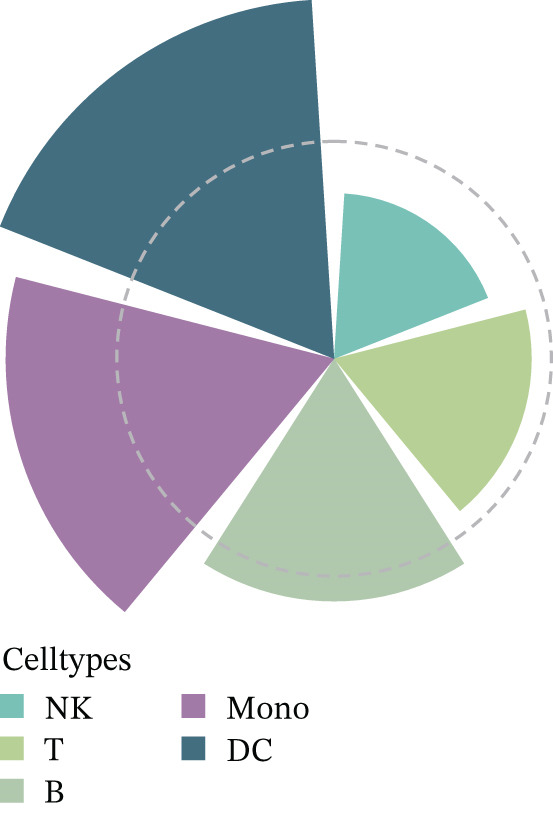
(h)

**Figure:**
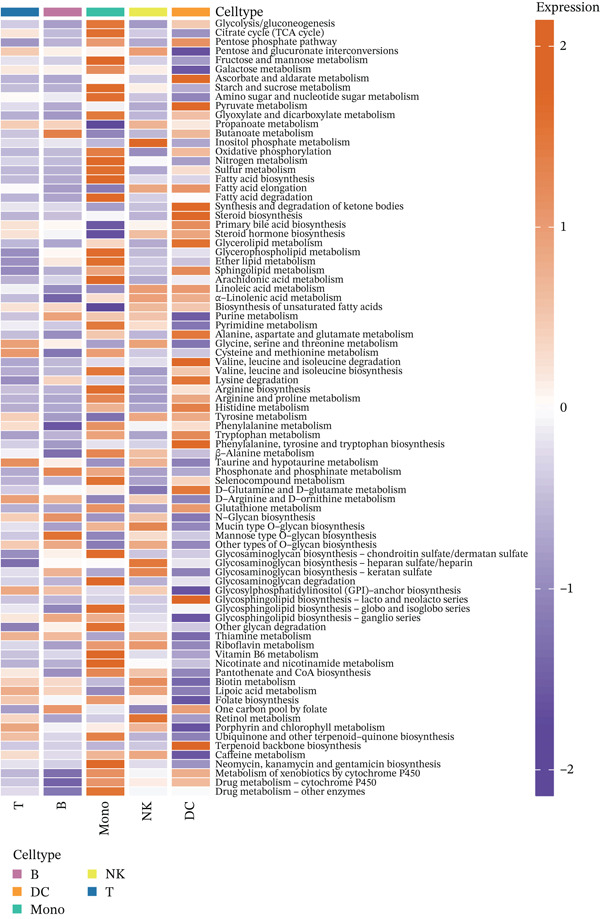
(i)

Further investigation of the Top 10 DEGs in T cells revealed their significant involvement in various inflammation‐ and immune‐related pathways, including the NOD‐like receptor signaling pathway and the T cell receptor signaling pathway (Figure 3c). We identified 3976 BPs (Figure 3d) and 428 KEGG signaling pathways (Figure 3e) involved in the five major cell types. Key BPs significantly enriched in each cell type include: T cells were related to canonical Wnt signaling pathway and cellular component disassembly; NK cells were involved in NK cell mediated cytotoxicity and regulation of myeloid cell differentiation; Mono was mainly involved in gamma‐delta T cell activation and pathways involved in phagocytosis; DC was significantly involved in both positive regulation of cellular component biogenesis and B cell proliferation; and B cells were involved in chromatin remodeling and regulation of translational initiation. Furthermore, five pathways were significantly enriched across multiple cell types: T cells were mainly involved in Wnt and sphingolipid signaling pathways; NK cells were significantly involved in neurotrophin signaling pathway, Th1 and Th2 cell differentiation; Mono was significantly involved in apoptosis and antigen processing and presentation; DC significantly participated in ferroptosis and transcriptional misregulation in cancer; and B cells were mainly associated with the oxytocin and relaxin signaling pathways. These results suggest that MI pathological process involves the coordinated action of multiple cell types and their specific BPs and pathways. Our study provides important insights into the immune regulatory mechanisms of MI and the development of targeted therapies.

Based on the CellChat analysis, we identified significantly activated ligand‐receptor pairs in MI (Figure 3f), with Mono playing a dominant role in the intercellular communication network (Figure 3g). Further analysis revealed that DC and Mono contributed the highest to MI pathological process (Figure 3h), suggesting that both cell types may contribute to MI′s immune regulation. Notably, among the five cell types, the significantly overexpressed metabolic pathways were mainly concentrated in Mono (Figure 3i), including glycolysis/gluconeogenesis, which indicated involvement in MI pathological process through metabolic reprogramming.

### 3.3. T Cell Transcriptomics Reveals Shared and Specific Immune Mechanisms in MI, MD, and VM

In the T cell transcriptomic analysis, we systematically compared the DEGs between the HC group and the three disease groups (MI, MD, and VM), where 914 DEGs were screened, including 157 upregulated and 757 downregulated genes (Figure 4a). In the HC and MD groups, 787 DEGs were screened, including 103 upregulated and 684 downregulated genes (Figure 4b). In the HC and VM groups, 988 DEGs were screened, containing 70 upregulated and 918 downregulated genes (Figure 4c). These findings indicate that T cells exhibit significant transcriptomic heterogeneity across different disease states; hence, they may perform different immune regulatory functions in MI, MD, and VM.

Figure 4Differential gene expression and functional roles of intercellular genes in migraine. (a) Volcano plot of differentially expressed genes (DEGs) between the healthy and migraine groups. (b) Volcano plot showing the distribution of DEGs between the healthy and Ménière′s disease groups (blue: upregulated, red: downregulated). (c) Volcano plot of DEGs between the healthy and vestibular migraine groups (blue: upregulated, red: downregulated). (d) Venn diagram of intersecting DEGs across the three disease groups. (e) Functional enrichment analysis of the intersecting DEGs. (f) Pathway enrichment analysis of the intersecting DEGs. (g) Enrichment network diagram of pathway correlations. (h) Gene set enrichment analysis of shared pathways.

**Figure:**
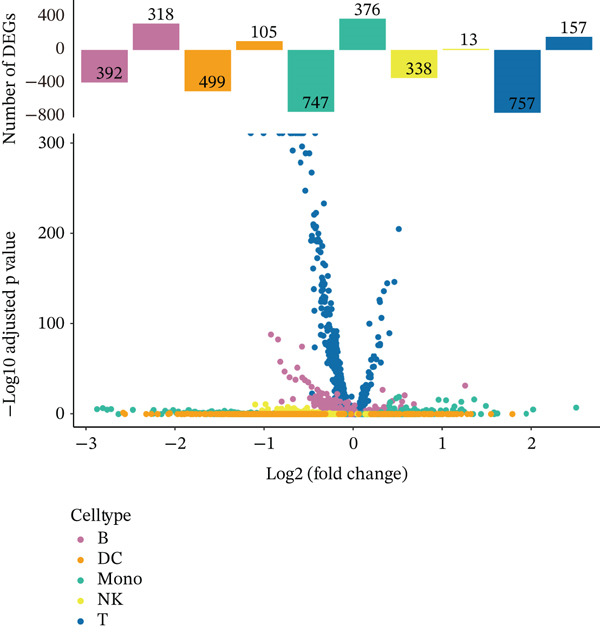
(a)

**Figure:**
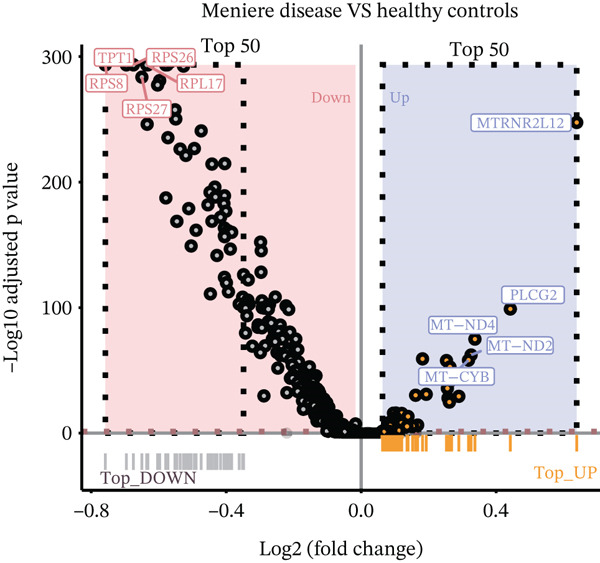
(b)

**Figure:**
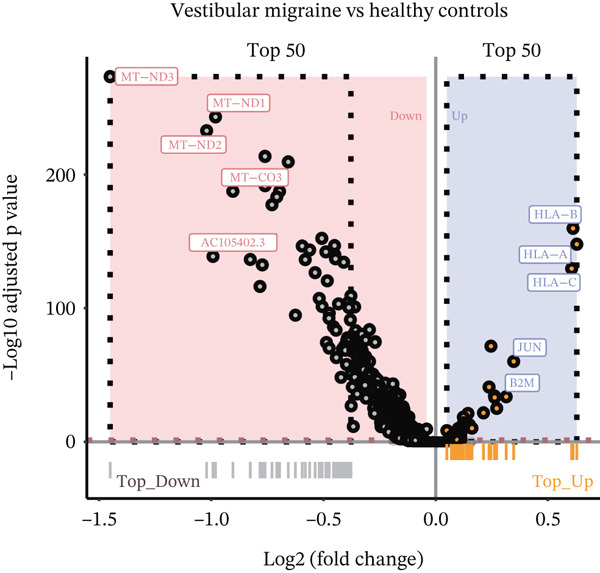
(c)

**Figure:**
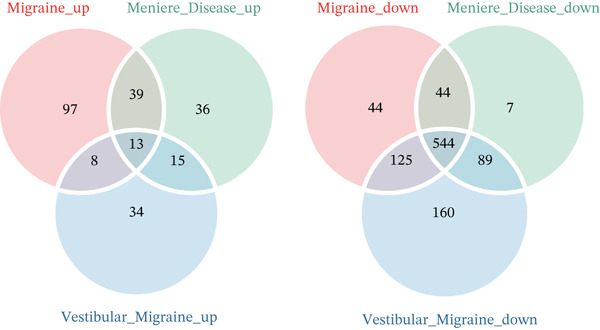
(d)

**Figure:**
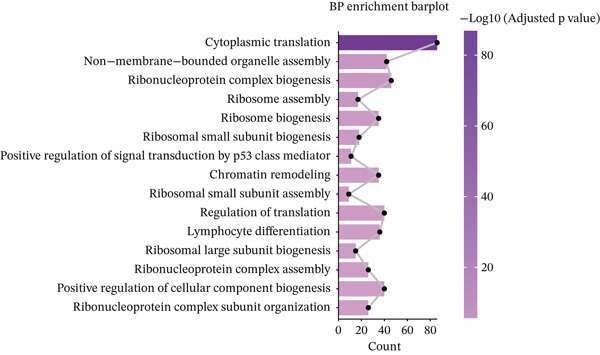
(e)

**Figure:**
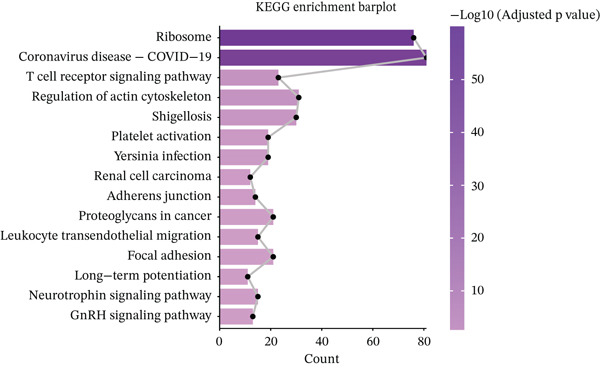
(f)

**Figure:**
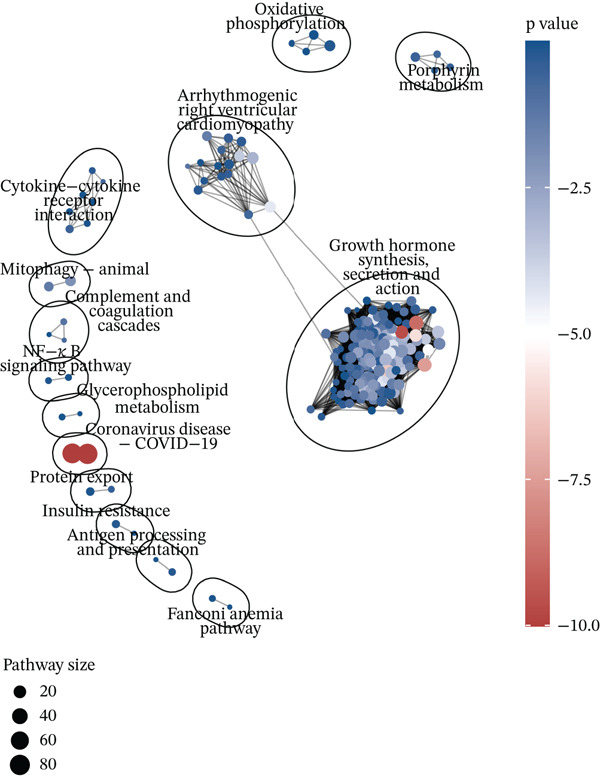
(g)

**Figure:**
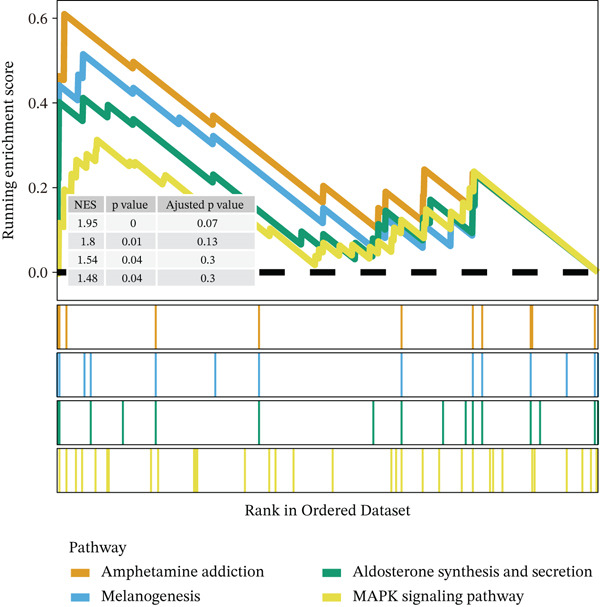
(h)

Notably, through Venn diagram analysis of the DEGs, we identified 557 intersecting DEGs (Figure 4d), of which 13 were consistently upregulated and 544 showed a consistent downregulation trend. Functional enrichment analysis of intersecting DEGs showed their significant involvement in 569 BPs (Figure 4e), including cytoplasmic translation and translation regulation, suggesting that common molecular mechanisms across diseases may involve the coordinated regulation of protein synthesis pathways. Furthermore, pathway analysis revealed that these intersecting DEGs were significantly involved in 63 pathways (Figure 4f), including the T cell receptor signaling pathway and ribosome. Further, we constructed an enrichment network graph that displayed the correlations between pathways (Figure 4g). Notably, among the DEGs, intersecting genes, and pathways jointly involved in the GSEA of MI cells, we identified four significantly activated intersecting pathways: amphetamine addiction, melanogenesis, aldosterone synthesis and secretion, and the MAPK signaling pathway (Figure 4h). Of these, the MAPK signaling pathway, which involved the most genes, was selected for in‐depth analysis.

### 3.4. MAPK Signaling Pathway Regulates Immune Cell Function and Identification of Key T Cell Genes in MI

Previous studies have shown that MI onset is closely related to abnormal activation of the MAPK signaling pathway [26]. Using a Sankey diagram displaying gene correlations between the MAPK signaling pathway and different cell types, we revealed the MAPK pathway′s regulatory role in B cells, DCs, NK cells, and T cells (Figure 5a). MAPK signaling pathway showed the strongest correlation with T and B cells, suggesting its primary involvement in regulating adaptive immune responses. Conversely, the MAPK pathway exhibited a moderate correlation with DCs and NK cells; hence, it is important in innate immune responses. These findings suggest that the MAPK signaling pathway has broad immune and inflammatory functions by regulating the activation, differentiation, and function of various immune cells.

Figure 5Core MAPK signaling pathway genes expression and target‐herbs prediction. (a) Sankey diagram of MAPK gene expression across different cell types. (b) Density plot showing the expression levels of the Top 10 genes of the MAPK signaling pathway in T cells. (c) Gene interaction network for four activated pathways (node size: gene expression; color: pathway). (d) Network diagram of herbs (circular), herbal ingredients (triangular), and targets (square). The degree of a node is defined as the number of its connected edges. The weight represents the connection time of the relationship. (e) Herbs, herbal components, and targets node connectivity ranking in network construction. A higher degree value indicates stronger connections between them. Red for herbs, green for compounds, and blue for targets. (f) Sankey diagram of herbs, herbal components, and targets, the visualization shows how multicomponent herbs might collectively modulate the disease network. The width of the bands is proportional to the number or strength of associations. DC, dendritic cell; Mono, monocyte; NK, natural killer.

**Figure:**
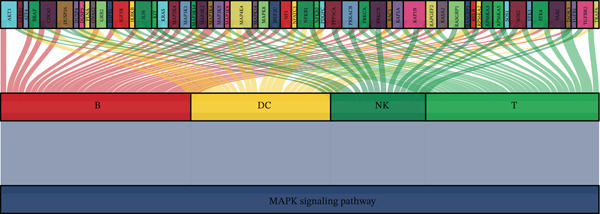
(a)

**Figure:**
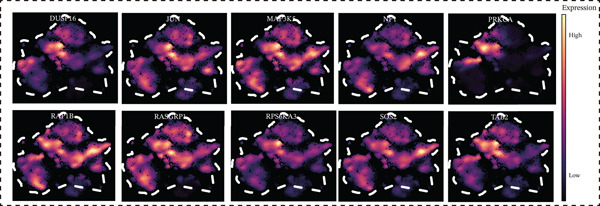
(b)

**Figure:**
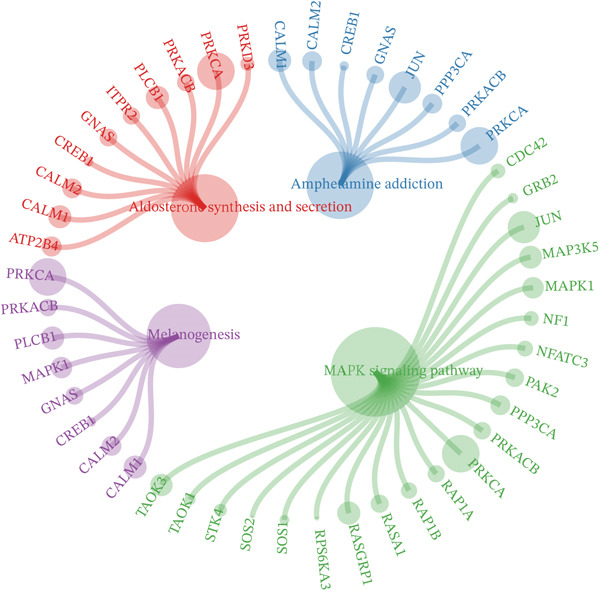
(c)

**Figure:**
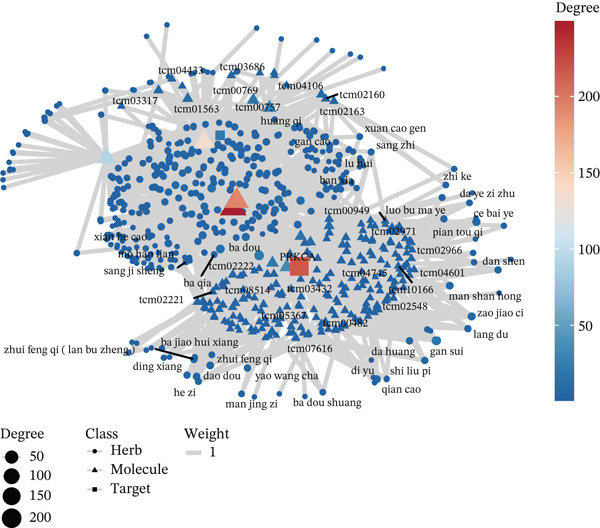
(d)

**Figure:**
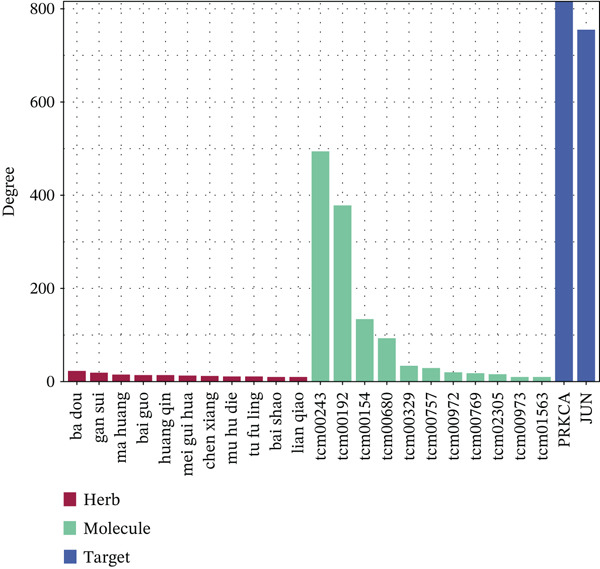
(e)

**Figure:**
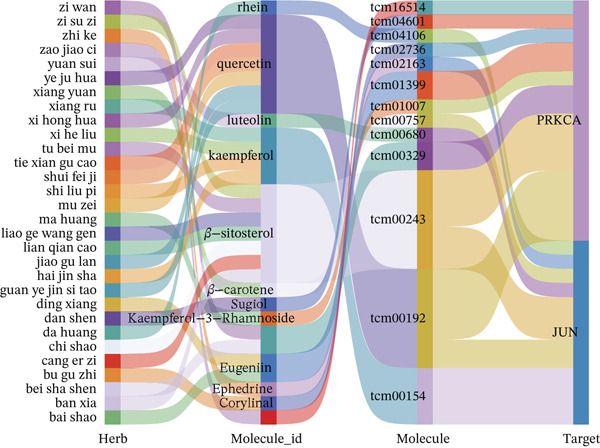
(f)

To further investigate the key molecular changes in T cells during MI, we analyzed the Top 10 MAPK signaling pathway‐related genes in T cells (Figure 5b), including *DUSP16*, *JUN*, *MAP3K5*, *NF1*, *PRKCA*, *RAP1B*, *RASGRP1*, *RPS6KA3*, *SOS2*, and *TAB2*. We also correlated four significantly activated intersecting pathways with their related genes (Figure 5c). Among the Top 10 expressed genes in T cells, *JUN* and *PRKCA* showed significant upregulation in the MI group compared with the HC group (Figure S1). These genes are potential therapeutic targets for MI; however, their mechanisms require further investigation.

### 3.5. Prediction of Target–Drug Interaction in MI

In Figure 5d, 1571 interaction pairs were screened between *JUN* and *PRKCA* matches for 433 herbs and 234 molecules. We further displayed the node connectivity ranking of the herbs (ba dou and gan sui), molecules (tcm00243 and tcm00192), and targets (*JUN* and *PRKCA*) network (Figure 5e). Finally, a Sankey diagram shows the interplay between herb, molecule, and target, which reveals complex network regulation (Figure 5f). In summary, herbs such as ba dou and gan sui exert their effects through key active components like tcm00243 and tcm00192, synergistically acting on core targets including *JUN* and *PRKCA*, thereby forming a complex multicomponent, multitarget network regulatory system that reveals their potential mechanism of action.

### 3.6. MAPK Signaling Pathway Activation in T Cell Subtypes

Using NMF analysis of 33,977 T cells from PBMC samples, we identified nine metagene profiles potentially corresponding to distinct T cell subtypes (Figure 6a). As shown in Figure 6b,c, we performed t‐SNE dimensionality reduction, identifying 11 cell clusters classified into four T cell subtypes: CD4^+^ T (*TCF7* and *LEF1*), CD8^+^ T (*CD8A* and *GNLY*), Treg (*IKZF2* and *ITGAE*), and naïve T (*CD2* and *IL7R*) cells. As shown in Figure 6d, the proportion of Treg and CD4^+^ T was increased significantly in the MD, VM, and MI groups compared with the HC group. Additionally, the Top 10 genes were highly expressed in T cell subtypes, indicating their functional role in regulating specific T cell subtypes (Figure 6e). Gene set activity scoring revealed a significantly high activity of the MAPK signaling pathway in T cells, hence, important for the regulation of T cell functions (Figure 6f). In addition, the significant activation of the MAPK signaling pathway was observed across all groups (Figure 6g). Compared with the HC group, we observed significant upregulation in the MI group, whereas the MD and VM groups showed significant downregulation (Figure 6h), indicating their important role in T cell function regulation and disease progression.

Figure 6T cell subtype characteristics and MAPK signaling pathway activity. (a) Nonnegative matrix factorization analysis of 33,977 T cells from blood samples, with color intensity indicating the strength of NMF factor expression. (b) t‐Distributed stochastic neighbor embedding clustering of T cells into 11 clusters and four subtypes. (c) Bubble diagram showing the known marker genes of T cells. (d) Abundance changes of T subtypes across different groups. (e) Top 10 gene expression in T cell subtypes (small density plot). (f) MAPK pathway activity in T cell subtypes (density plot; blue: low, green: high). (g) Density chart showing the overall activity of the MAPK signaling pathway across all groups. (h) Density chart showing the activation status of the MAPK signaling pathway in each group. MP, metagene profiles; t‐SNE, t‐distributed stochastic neighbor embedding.

**Figure:**
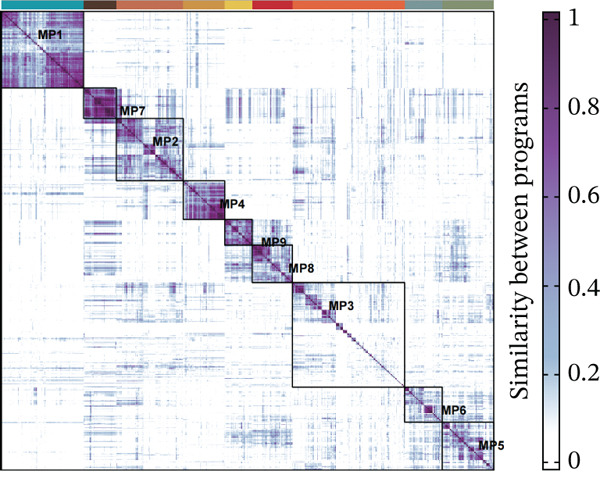
(a)

**Figure:**
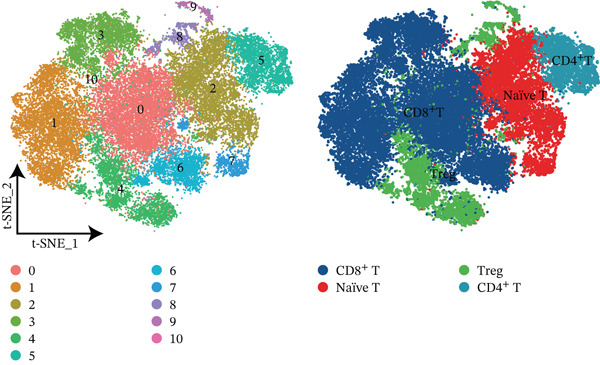
(b)

**Figure:**
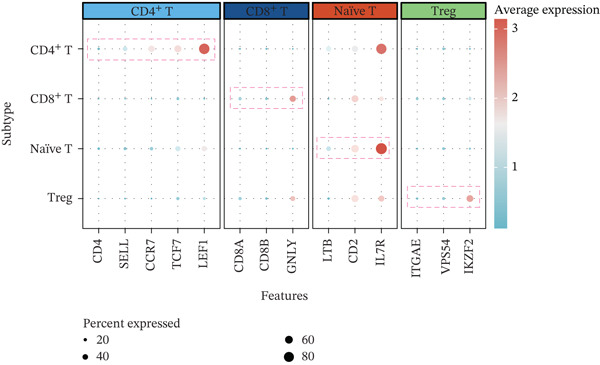
(c)

**Figure:**
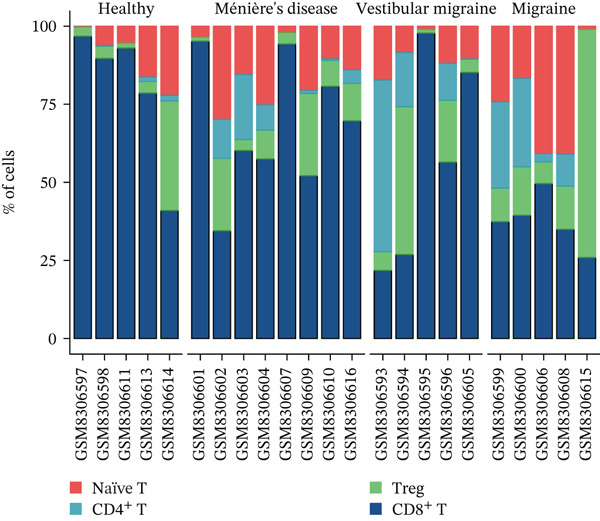
(d)

**Figure:**
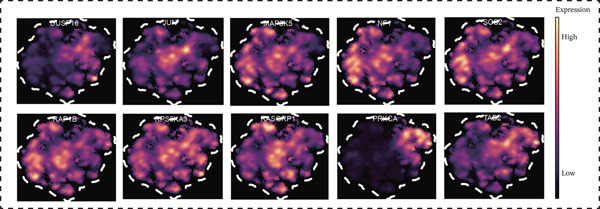
(e)

**Figure:**
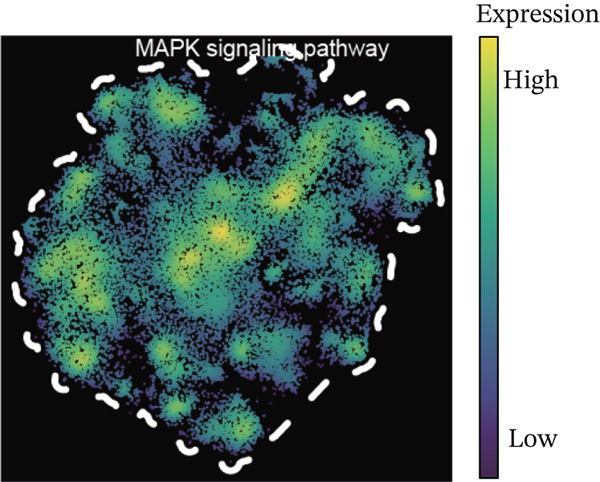
(f)

**Figure:**
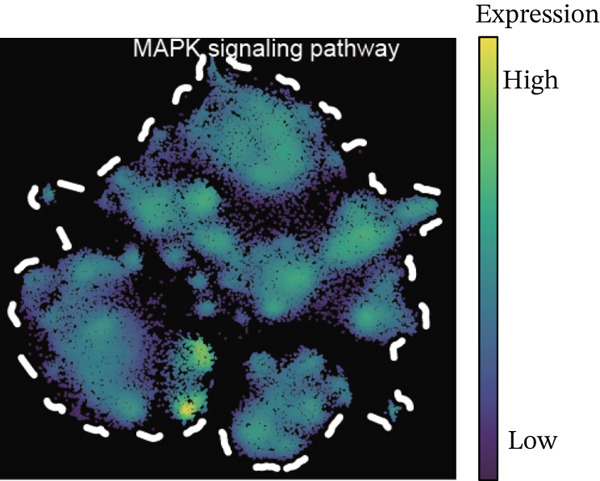
(g)

**Figure:**
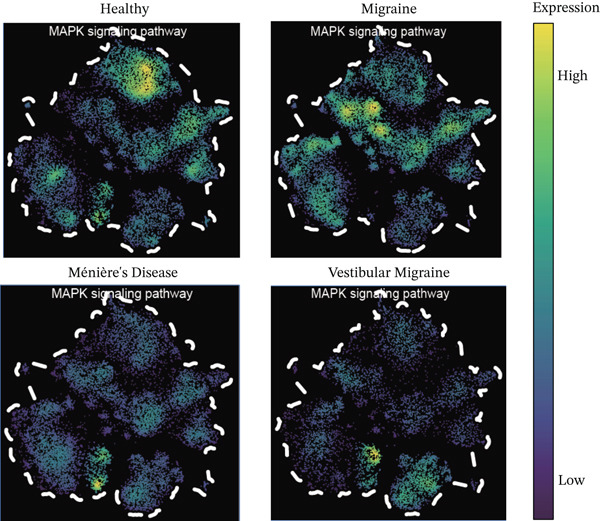
(h)

### 3.7. PRKCA‐Mediated T Cell Differentiation Trajectory Reprogramming in MI and Related Diseases

Based on the trajectory inference revealed two distinct differentiation paths: one from CD8^+^ T cells to CD4^+^ T cells and the other toward naïve T cells (Figure 7a). Distribution characteristics of these cell subtypes showed that naïve T cells and Tregs were predominantly enriched along Trajectory 1, whereas CD4^+^ T cells significantly accumulated along Trajectory 2 (Figure 7b). Notably, cells from the three disease types exhibited heterogeneous distribution characteristics along the differentiation trajectories (Figure 7c). The key regulatory genes, *MAP3K5* and *PRKCA*, displayed dynamic expression patterns during the differentiation process. *MAP3K5* was downregulated in both trajectories, whereas *PRKCA* expression significantly increased with pseudotime progression (Figure 7d). Compared with the HC group, *PRKCA* was significantly upregulated in all three disease groups, whereas *MAP3K5* maintained baseline expression levels in both the HC and the three disease groups (Figure 7e). The two T cell differentiation trajectories, starting from CD8^+^ T cells, revealed that *PRKCA* was significantly upregulated in patients with MI, MD, and VM, suggesting that PRKCA may be a hub gene that mediates T cell differentiation trajectories and plays a potential regulatory role in disease progression.

Figure 7T cell differentiation trajectory and key regulatory gene analysis. (a) Single‐cell trajectory analysis revealing two main paths of T cell differentiation. (b) Cell subtype distribution in differentiation trajectories. (c) Group distribution (migraine, Ménière′s disease, vestibular migraine, and healthy) in differentiation trajectories. (d) Dynamic expression patterns of key regulatory genes (*MAP3K5* and *PRKCA*) during differentiation. (e) *MAP3K5* and *PRKCA* expression in healthy and disease groups (violin plot). t‐SNE, t‐distributed stochastic neighbor embedding.

**Figure:**
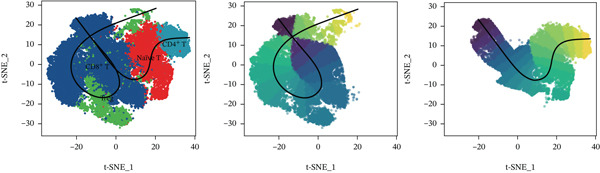
(a)

**Figure:**
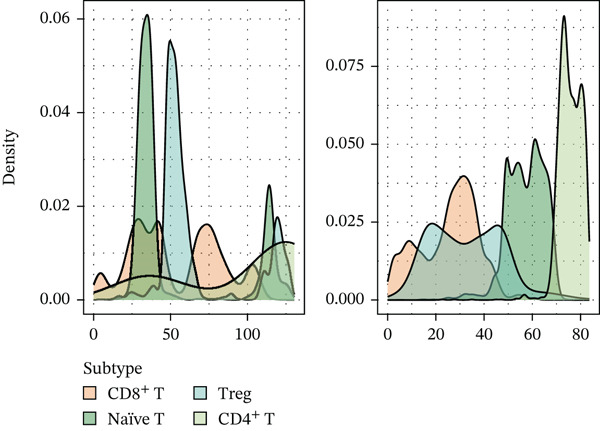
(b)

**Figure:**
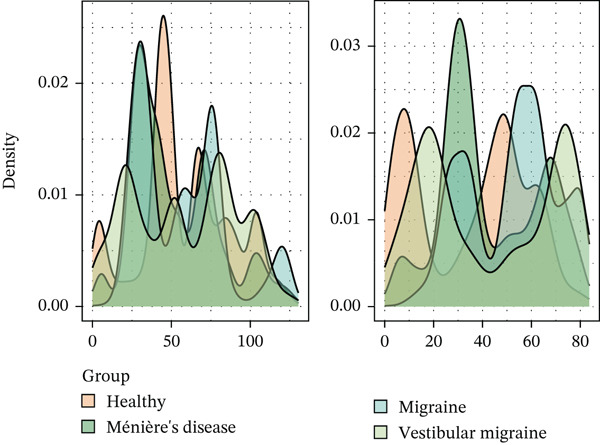
(c)

**Figure:**
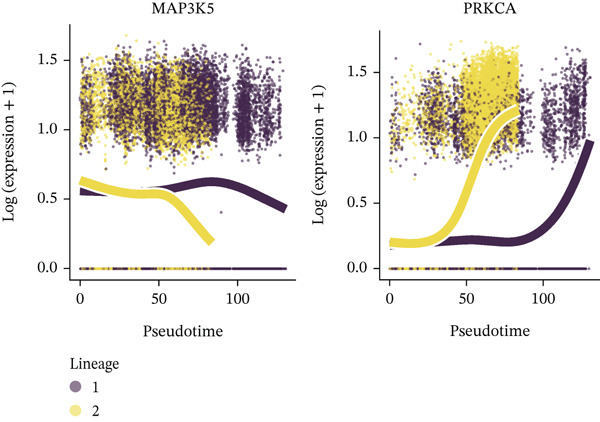
(d)

**Figure:**
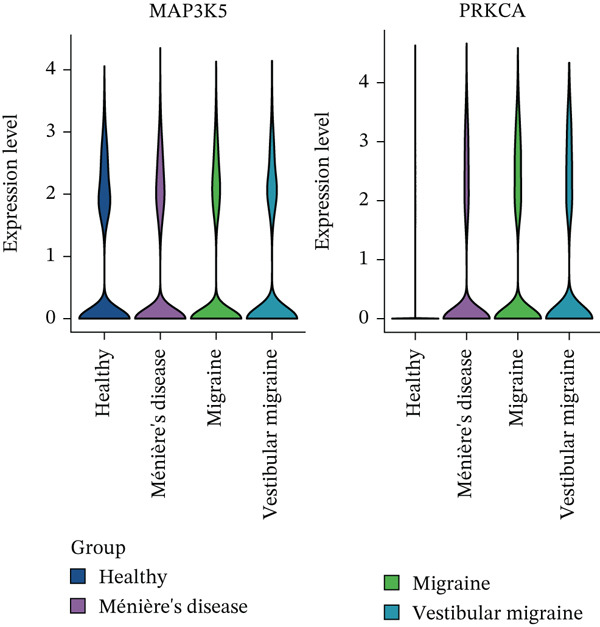
(e)

## 4. Discussion

MI, a complex neurological disorder, serves as a link between VM and MD [27]. Thus, a systemic inflammatory state may be involved in MI pathogenesis [10]. However, studies on the role of peripheral blood immune cells in MI pathophysiology are limited. Therefore, this study utilized scRNA‐seq technology to systematically analyze the immune landscape of PBMCs from patients with MI, MD, and VM, thereby revealing T cells′ role in disease progression and their potential molecular mechanisms. We identified the significance of T cell enrichment in the MI group and, through differentiation trajectory analysis and key gene expression patterns, clarified the potential regulatory roles of immune cell heterogeneity, metabolic reprogramming, and *PRKCA* in disease progression.

A key distinction lies in the role of Mono. Specifically, prior research by López‐Escánez has reported elevated levels of Mono‐derived proinflammatory cytokines, such as IL‐1*β*, in MD compared with VM [16]. Our scRNA‐seq data provide direct evidence of the degree of high infiltration of Mono in MD patients. In stark contrast, the immune landscape of MI was characterized by a lymphoid‐centric response. Specifically, consistent with the results of a previous study [28], T cell abundance had a significant increase in the MI group compared with the HC group, whereas the abundance of Mono was significantly decreased. This finding aligned with those of previous studies suggesting that T cells impact the MI pathological process through neuroinflammation, immune dysregulation, and cell–cell interactions [25]. Other studies have indicated that Mono, in patients with MI, may contribute to neuroinflammatory responses by secreting proinflammatory cytokines, thereby exacerbating headache symptoms [29]. These findings collectively highlight immune cells′ critical role in MI, providing important clues for further understanding its pathological mechanisms.

CellChat analysis revealed that Mono and DC dominate the cell–cell communication network in MI. We found that Mono underwent substantial metabolic reprogramming, characterized by the significant enrichment of the pentose phosphate and glycolysis pathways. Under hypoxic microenvironmental stimulation, immune cells establish a novel immune‐metabolic coupling pattern through metabolic reprogramming, thus maintaining their inflammation‐regulatory capacity [30]. The significant enrichment of the pentose phosphate and glycolysis pathways implicated a pivotal role of energy metabolism in MI [31]. This metabolic adaptation may elicit MI′s pathological progression. Metabolic reprogramming plays a key role in inflammation regulation and offers a potential therapeutic target for MI, though further research is needed to uncover the underlying mechanisms [32]. In this context, DCs drive immune synapse formation of T cells in the central nervous system, triggering an activation cascade of T lymphocytes that exacerbates the local inflammatory response [33]. Therefore, MI′s pathological process involves the complex regulation of immune cell metabolic reprogramming, cell–cell communication, and inflammatory responses.

The MAPK signaling pathway was significantly upregulated in the MI group and downregulated in the MD and VM groups. This contrasting pattern may reflect the distinct disease mechanisms, whereas MI is primarily driven by central neuronal sensitization and facilitated by MAPK activation; the downregulation in MD and VM could indicate a predominant anti‐inflammatory or immunosuppressive state in the peripheral immune compartment. The MAPK pathway is a highly conserved kinase response system [34]. Inhibition of this pathway alleviates inflammation and neuropathic pain in animal models [35]. Furthermore, abnormal activation can sensitize nociceptors and induce synaptic plasticity, contributing to neuropathic and inflammatory pain [36]. Notably, recent research has confirmed that MAPK pathway inhibitors can effectively alleviate trigeminal neuralgia pain conduction, providing an important theoretical basis for the development of new analgesic therapies [37]. Targeted regulation of the MAPK signaling pathway could be a potential therapeutic strategy for treating MI and its related diseases.

Pseudotime analysis reconstructed the developmental trajectory of T cells, revealing a significant multipotent differentiation potential in a subset of CD8^+^ T cells. These cells demonstrated a capacity to transition towards states resembling CD4^+^ T cells or revert to naïve T cells, underscoring a high degree of plasticity in the T cell differentiation of these patients. Notably, during the differentiation process, *PRKCA* showed a significant upward trend, with high expression in the MI, MD, and VM groups. Previous studies have confirmed that *PRKCA* polymorphisms promote MI occurrence and development by regulating the release of inflammatory factors [26]. *PRKCA* can protect epigallocatechin gallate against lipopolysaccharide‐induced inflammation by inhibiting the release of inflammatory factors via the MAPK signaling pathway [38]. Further studies revealed that *JUN* and *PRKCA* exhibited significantly higher expression in the MI group. *JUN*, a core member of the AP‐1 transcription factor family, can cause neuronal axon damage [39]. Research on *JUN* in MI, MD, and VM is limited. Given the connection between *PRKCA*, MAPK signaling, and cytokine regulation, future studies should investigate whether this pathway contributes to the differential cytokine signatures observed between VM and MD by López‐Escánez [16].

Importantly, our integrated network pharmacology analysis offers a direct translational perspective by identifying potential herbal compounds that could target these key molecules. Herbal compounds (ba dou and gan sui) and their active components (tcm00243) are predicted to synergistically act on *JUN* and *PRKCA* core targets. Both *JUN* and *PRKCA* are critical molecules closely associated with cell proliferation and inflammation and are highly correlated with MI pathology. Therefore, these predicted core targets provide crucial theoretical foundations and specific molecular markers for further elucidating the precise mechanisms of this compound and developing targeted intervention strategies.

Although this study revealed immune microenvironment reprogramming in MI and related diseases, limitations include the relatively small sample size. Secondly, the analysis was confined to peripheral immune cells, and the lack of central nervous system samples restricted direct investigation of neuroimmune interactions. Thirdly, the unavailability of clinical metadata constrains our analysis by preventing adjustment for confounders. Thus, despite identifying significant immune variations, the influence of missing variables cannot be determined, highlighting a necessity for incorporating such data in future studies. Finally, the bioinformatically identified roles of *PRKCA* and *JUN* in the MAPK pathway call for experimental validation using molecular methods like gene knockdown or reporter assays. Consequently, future research should prioritize larger, clinically annotated cohorts, incorporate brain tissue where possible, and employ molecular experiments to mechanistically confirm the roles of key targets like *PRKCA* and *JUN*.

## 5. Conclusions

Using single‐cell transcriptomics, this study systematically analyzed the immune landscapes of MI, MD, and VM, revealing the central role of T cells and their potential molecular mechanisms in disease development. *PRKCA*‐mediated T cell differentiation trajectory reprogramming and abnormal MAPK signaling pathway activation may be important pathological features of these diseases. These findings provide a foundation for understanding immune regulatory mechanisms and developing targeted therapies.

## Author Contributions

All authors contributed to the study conception and design. Mika Pan, Liyan Lu, Wenyi Song, Qiling Wan, Li Su, and Donghua Zou conceived and designed the experiments; Mika Pan, Li Su, Qi Huang, Guining Liang, Qingyan Wei, Wenyi Song, Jingyi Zeng, Yating Lan, Chun Zou, and Youfeng Xie collected, analyzed and interpreted the data; Mika Pan, Liyan Lu, Wenyi Song, Guining Liang, and Qingyan Wei wrote the original draft; Qiling Wan, Li Su, and Donghua Zou reviewed and edited the draft. Mika Pan, Liyan Lu, and Wenyi Song authors contributed equally to this work and share first authorship.

## Funding

This study was supported by the Joint Project on Regional High‐Incidence Diseases Research of Guangxi Natural Science Foundation (2024GXNSFDA010001); the Youth Science Foundation of Guangxi Medical University (GXMUYSF202534); the Joint Project of Youth Science Foundation of Guangxi Medical University (GXMUYSFB2026043 and GXMUYSFB2026087); the Scientific Research Project of Guangxi Health Commission (Z‐A20250579 and Z‐A20250588); the Key Talent Program of Guangxi Zhuang Autonomous Region (Bagui Young Excellence Talents to Donghua Zou); First‐Class Discipline Innovation‐Driven Talent Program of Guangxi Medical University; and Guangxi Medical and Health Key Discipline Construction Project.

## Disclosure

All authors have read and agreed to the published version of the manuscript.

## Ethics Statement

Ethical approval was not required for this study because it did not involve any human experiments.

## Consent

The authors have nothing to report.

## Conflicts of Interest

The authors declare no conflicts of interest.

## Supporting information


**Supporting Information** Additional supporting information can be found online in the Supporting Information section. Figure S1: *JUN* and *PRKCA* expression in T cell subtypes. Violin plot shows comparison between healthy and migraine groups.

## Data Availability

Data were downloaded from the public database online at the GEO database under Accession Number GSE269117.
